# Patient navigator team perceptions on the implementation of a citywide breast cancer patient navigation protocol: a qualitative study

**DOI:** 10.1186/s12913-022-08090-3

**Published:** 2022-05-21

**Authors:** Stephanie Loo, Katelyn Mullikin, Charlotte Robbins, Victoria Xiao, Tracy A. Battaglia, Stephenie C. Lemon, Christine Gunn, the TRIP Consortium

**Affiliations:** 1 Department of Health Law, Policy, and Management, School of Public Health, Boston University, 715 Albany St, Boston, MA 02118 USA; 2 Women’s Health Unit, Section of General Internal Medicine, Evans Department of Medicine, Boston Medical Center and Boston University School of Medicine, Boston, MA USA; 3 University of Massachusetts Chan Medical School, Worcester, MA USA; 4 The Dartmouth Institute for Health Policy and Clinical Practice and Dartmouth Cancer Center, Geisel School of Medicine, Dartmouth College, Lebanon, NH USA

**Keywords:** Patient navigation, Breast cancer care, Social risk screenings, CFIR

## Abstract

**Background:**

In 2018 Translating Research Into Practice (TRIP), an evidence-based patient navigation intervention aimed at addressing breast cancer care disparities, was implemented across six Boston hospitals. This study assesses patient navigator team member perspectives regarding implementation barriers and facilitators one year post-study implementation.

**Methods:**

We conducted in-depth qualitative interviews at the six sites participating in the pragmatic TRIP trial from December 2019 to March 2021. Navigation team members involved with breast cancer care navigation processes at each site were interviewed at least 12 months after intervention implementation. Interview questions were designed to address domains of the Consolidated Framework for Implementation Research (CFIR), focusing on barriers and facilitators to implementing the intervention that included 1) rigorous 11-step guidelines for navigation, 2) a shared patient registry and 3) a social risk screening and referral program. Analysis was structured using deductive codes representing domains and constructs within CFIR.

**Results:**

Seventeen interviews were conducted with patient navigators, their supervisors, and designated clinical champions. Participants identified the following benefits provided by the TRIP intervention: 1) increased networking and connections for navigators across clinical sites (*Cosmopolitanism*), 2) formalization of the patient navigation process (*Goals and Purpose, Access to Knowledge and Information, and Relative Advantage*), and 3) flexibility within the TRIP intervention that allowed for diversity in implementation and use of TRIP components across sites (*Adaptability*). Barriers included those related to documentation requirements (*Complexity*) and the structured patient follow up guidelines that did not always align with the timeline of existing site navigation processes (*Relative Priority*).

**Conclusions:**

Our analysis provides data using real-world experience from an intervention trial in progress, identifying barriers and facilitators to implementing an evidence-based patient navigation intervention for breast cancer care. We identified core processes that facilitated the navigators’ patient-focused tasks and role on the clinical team. Barriers encountered reflect limitations of navigator funding models and high caseload.

**Trial registration:**

Clinical Trial Registration Number NCT03514433, 5/2/2018.

## Background

Breast cancer disparities have been well-established in the literature. Black women experience the highest mortality rate of all racial and ethnic groups at 27.1 deaths per 100,000 people; nearly two-times greater breast cancer-specific mortality compared to white women [1, 2]. Disparities in timely diagnosis, treatment, and outcomes have also been shown in Hispanic/Latina women, those who rely on Medicaid/Medicare, and those whose primary language is not English [3–8]. Patient navigation is an evidence-based intervention that is promoted by the American College of Surgeons, which requires accredited breast cancer care sites to have established patient navigator programs [9].

Patient navigation promotes timely access to diagnosis and treatment by eliminating barriers to care via one-on-one interactions between patients and navigators [10, 11]. While qualifications and models of navigation vary across health systems [12], navigators are meant to work within health care systems to identify and resolve barriers to timely receipt of care and other support services for individual patients. Breast cancer care has been one area with known disparities where patient navigation interventions have demonstrated a positive impact on reducing times to diagnosis [13] and treatment [7, 14–16]. Prior research examining patient navigation in cancer care has identified high patient and provider satisfaction with patient navigation programs along with reductions in the time interval between biopsy and first consultation and time to initiation of treatment following a positive diagnosis [17].

There remains a need to study and evaluate patient navigation interventions, given the known variation in tasks that navigators assist with [18, 19], and increasing use of patient navigation programs across a multitude of primary and specialty care disciplines. More data are needed regarding the best practices of navigation in cancer care, identifying barriers and facilitators to the implementation of such practices, as well as how to scale up navigation to increase patient access to effective, evidence-based navigation services. This study seeks to build upon prior evaluation work of patient navigation interventions by assessing patient navigator and clinical team perspectives regarding barriers and facilitators to implementing an evidence-based breast cancer patient navigation intervention across six academic hospitals in Boston, MA [20].

## Methods

### Setting

**Fig. 1:**
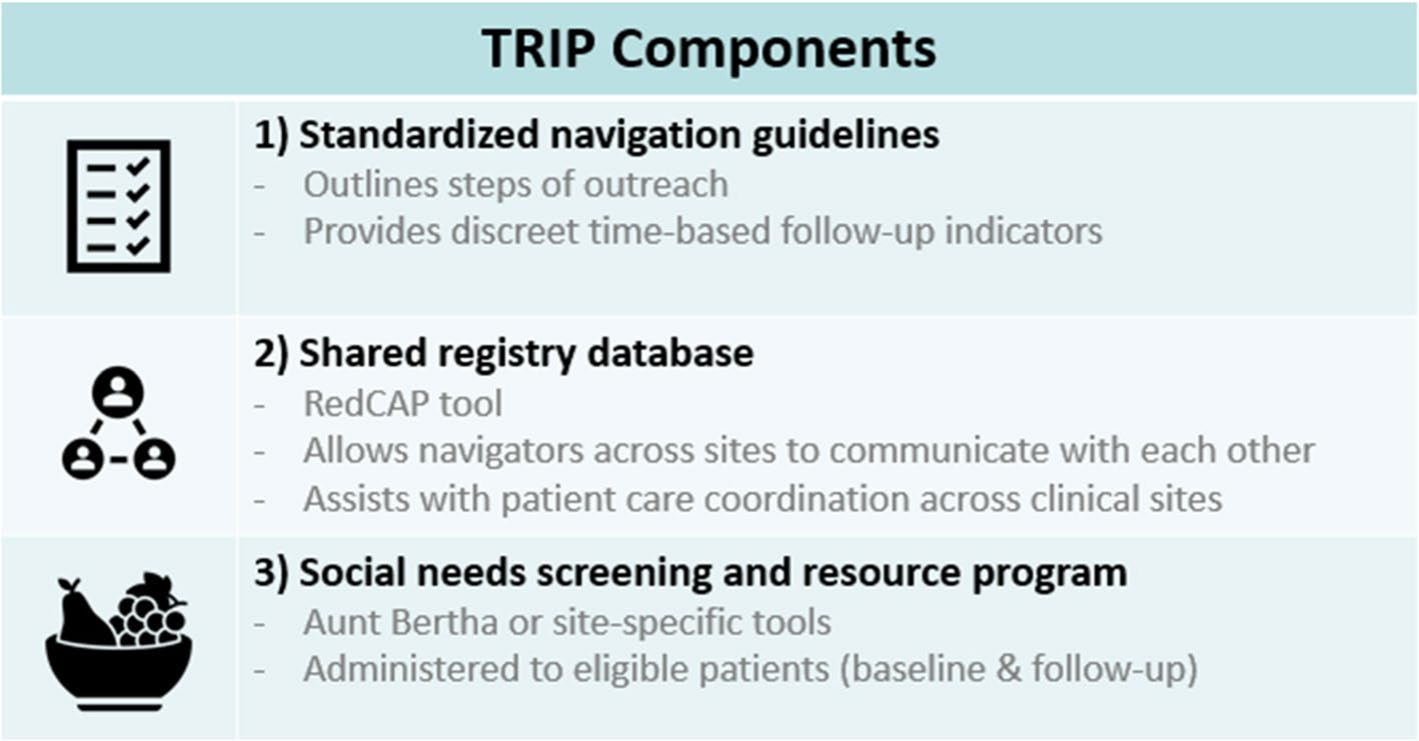
TRIP Intervention Components

Translating Research Into Practice (TRIP) is a cluster-randomized, stepped-wedge hybrid type I effectiveness-implementation trial, launched in 2018 across six breast cancer care clinics. Details of the TRIP study design and intervention have been published elsewhere [21]. The TRIP study is designed to evaluate the impact of a multi-level patient navigation intervention on timely, quality breast cancer care, with implementation data providing context to our main clinical outcome. Targeted enrollment for TRIP exceeds 1,000 patients across the six sites. The TRIP patient navigation intervention aims to reduce healthcare disparities for women who have been diagnosed with breast cancer, particularly women of color and those on public insurance. Navigators and their sites are tasked with implementing the three intervention components: 1) a navigation protocol consisting of an 11-step guideline to conducting evidence-based patient navigation in breast cancer care [11, 21], 2) a shared patient registry database for navigators to communicate with each other and coordinate patient care across clinical sites, and 3) implementation of systematic social needs screening and referral to resources using a web-based platform, with screening occurring at the time of diagnosis and 3-months follow-up (see Fig. 1). Prior to the implementation of TRIP, a rigorous assessment of current patient navigation activities at each participating site was conducted [22]. While navigation services were offered to all patients being treated for breast cancer, sites varied widely on how navigation was funded, patients served by their programs, and the type of services provided [22]. One site ceased providing breast cancer services during the study period and thus no longer contributed data to the study. This site is excluded from the implementation assessment presented here, as no interviews could be conducted. TRIP was implemented across sites in a stepped wedge format, with the program starting at a new site every three months beginning in September 2018 with the final site activated in November 2019.

### Recruitment and data collection

Core study implementers who had been employed at least six months at TRIP clinical sites who were invited to participate in a qualitative interview to learn about TRIP implementation. Core implementers included patient navigators, patient navigator supervisors and clinical champions at each site who were solicited for interviews by the central study team via email. Additional participants were recruited via snowball sampling via study participants who were asked to identify clinical team members who could speak to the TRIP implementation or patient navigation processes at their site. This study was reviewed and approved by the Boston Medical Center and Boston University Medical Campus Institutional Review Boards.

Interviews were conducted using a semi-structured, flexible interview guide designed to elicit descriptions of patient navigation processes at each clinical site, experiences with implementing TRIP, benefits and challenges of the TRIP program overall, and TRIP component-specific questions (e.g., use of the navigation guidelines, shared registry, and social needs screening tools). Questions addressed implementation of TRIP using the overall study’s guiding implementation framework, the Consolidated Framework for Implementation Research (CFIR) [23]. A priori expert consensus was reached within the TRIP investigative team on which CFIR constructs and domains were likely to be most relevant to TRIP barriers and facilitators. Interviewers were not part of the study implementation team and were experienced qualitative interviewers.

All interviews were conducted between December 2019 and March 2021, either in-person or via telephone in a private setting without other individuals present. Interviewees provided verbal informed consent prior to beginning interviews, and were provided compensation for participation. Interviews were audio recorded with the permission of the interviewee. Interviewers completed field memos following completion of interviews.

### Data analysis

All interviews were transcribed by a professional service and entered into Dedoose 9.0.18 to facilitate team coding and analysis. An analytical codebook using domains and constructs from the CFIR model was developed. The codebook included the CFIR domains of Implementation Climate, Individual Characteristics, Intervention Characteristics, Learning Climate, Outer Setting, and Readiness for Implementation, as these were the domains addressed in the interview guide (see Table 1 for Codebook and Definitions). The coding team (CG, SL, KM, and CR) conducted initial coding of all interviews under guidance of the qualitative lead (CG). Each interview was coded by at least two coders, with SL coding all interviews. The coding team met weekly to review coding and adjudicate differences in application of codes. Initially, each passage relevant to TRIP’s implementation was coded to a corresponding CFIR domain or construct. In a secondary phase, CG, SL, and CR reviewed each CFIR code to identify barriers and facilitators relevant to implementation and determine if the construct was adequately represented in the data. Resulting themes were discussed and finalized amongst the full research study team. Preliminary results were shared with the TRIP clinical advisory panel and program patient navigators for member checking and to bolster credibility of our findings.

**Table 1:** CFIR Domains, Constructs and Codebook Definitions

CFIR Domain	CFIR Construct	Operational Definition of CFIR Construct for Coding
**Implementation Climate**	Goals/Purpose	The extent to which participants felt that TRIP objectives were aligned or not aligned with goals/purpose of clinical site
	Relative Priority	Any descriptions of how important conducting TRIP activities (the three TRIP components) were at the site. This included perceptions of how much support there was to implement TRIP at the site and instances that show how TRIP was valued or de-valued related to other navigation activities or priorities
**Intervention Characteristics**	Adaptability	Perceptions of or examples of how the TRIP intervention can be or has been adapted, tailored, refined or reinvented to meet the needs of the local hospital
	Complexity	The perceived difficulty or ease of implementing the TRIP intervention at clinical sites, reflected by duration, scope, radicalness, disruptiveness, centrality, and intricacy and number of steps/components required to implement. There should be an evaluative statement about its complexity for something to be included here—not just its use
	Relative Advantage	Stakeholders’ perception of the advantage/disadvantage of implementing the TRIP intervention versus current or former practice within the clinic setting
**Outer Setting**	Cosmopolitanism	The degree to which individuals or the system are working with other sites to manage care for patients
**Readiness for Implementation**	Access to Knowledge & Information	The extent to which navigators feel training and other materials for the TRIP intervention are accessible, usable and useful

## Results

Nineteen individuals were approached for interviews with 17 agreeing to participate (89.5% recruitment rate). Seventeen interviews were conducted across five clinical sites in the Boston area, consisting of six breast cancer care patient navigators, three patient navigator supervisors, five clinical champions, and three clinical team members who worked closely with patient navigation processes (Table 2). Of the six navigators interviewed in our study, only one site utilized nurse navigators (*n*=2), the remaining were nonclinical, lay navigators who either were members of the local community or had experience working with community members. At the start of the study, all but one participating site had staff functioning in a navigator role, who agreed to adopt the TRIP protocol. The majority of participants were female (88%), White-identified (65%), and not Hispanic/Latino/a/x (71%). Participants had worked in their current roles for an average of 6.5 years.

**Table 2:** Sociodemographic Characteristics of Interview Participants (*N* = 17)

**Gender, N (%)**	
Female	15 (88.2)
Male	2 (11.8)
**Race, N (%)**	
American Indian or Alaskan Native	1 (5.9)
Asian	2 (11.8)
Black or African American	1 (5.9)
Caucasian or White	11 (64.7)
Native Hawaiian or Other Pacific Islander	1 (5.9)
Other	1 (5.9)
**Ethnicity, N (%)**	
Hispanic, Latino/a/x, or Spanish	4 (23.5)
Not Hispanic, Latino/a/x, or Spanish	12 (70.6)
Did not respond	1 (5.9)
**Clinic Role, N (%)**	
Patient Navigator Supervisor	3 (17.6)
Patient Navigator	6 (35.3)
Clinical Champion	5 (29.4)
Other Support Staff	3 (17.6)
**Years worked in clinic, mean (range)**	6.5 years (9 months-18 years)

The barriers and facilitators of the TRIP implementation identified were consistent with the following CFIR domains and accompanying constructs (Table 1): Implementation Climate: Goals/Purpose and Relative Priority; Intervention Characteristics: Adaptability, Complexity, and Relative Advantage; Outer Setting: Cosmopolitanism; and Readiness for Implementation: Access to Knowledge and Information. Our working definition for each construct were tailored to the TRIP project from the CFIR definitions described by Damschroder and colleagues [23].

### Implementation climate: goals/purpose

TRIP’s ease of adoption was related to the extent that TRIP was compatible with the site’s goals and purpose, as manifested through navigation processes, standards of care, or future site plans for navigation. TRIP was found to be widely accepted and compatible across participating clinical sites when this alignment of goals/purpose was present. Some respondents described the state of patient navigation at their clinical site prior to the implementation of TRIP:

*“I think [clinical site] has supported patient navigation ever since I’ve been [here]..[N]avigation has been, from our institutional standpoint, really important. ... I would say it’s part of the mosaic of what we do is to have the navigator there as part of the care facility.” Clinical Champion (CC)*

Having a strong clinical environment that was supportive of patient navigation activities predisposed sites to align with TRIP objectives and adopt the program without significant barriers. In addition to clinical buy-in, many participants noted that several activities included within the TRIP navigation protocol aligned with their on-going patient navigation work.

*“I don’t think that what [patients are] getting through TRIP is that different from what they got before. I think that it may be more streamlined... [b]ut I don’t think philosophically the program is that different.” Patient Navigator Supervisor (PNS)*

*“[T]hey have been easy to follow because they’re similar to what we would do for our patients who are not part of TRIP or who we don’t use the TRIP guidelines for.” Patient Navigator (PN)*

*“I think [TRIP is] doing what’s standard care. I think how they’ve done the study [by] collecting information from the patients or the patient navigator is what’s the standard of care at the hospital.” CC*

In some cases, the TRIP program aligned with on-going plans for the expansion of patient navigation services at a clinical site, whether that was an intentional structure to assess patients’ unmet social needs or expanding program services to different patient populations:

*“I think in many ways, TRIP is trying to achieve what I’ve tried to achieve, which is ... be mindful of the challenges that patients face as they go through the diagnosis and treatment of breast cancer, and incorporate in, at each of those junctures, a thoughtful appraisal of the social determinants of health, thinking about what are the specific challenges that specific patient may be experiencing, and then find solutions to make the journey a little bit easier.” PNS*

*“[TRIP] places more of an emphasis on the social determinants, in thinking about care beyond the scope of the immediate treatment that they’re receiving. You get a better understanding of the individual and their circumstances, and I think that enables me as an advocator to better support the patients that I work with.” PN*

Considering barriers related to the Goals/Purpose construct, one clinical site noted that the majority of breast cancer patients served did not align with the targeted patient population of the TRIP program. This resulted in difficulties in enrolling TRIP patients at this institution, which was perceived as a misalignment of the TRIP program’s goals regarding who the intervention was attempting to reach.

*“We see a lot of patients who are other types of minorities. TRIP was really focusing a lot on black patients, which we see proportionately fewer at [clinical site]. So since TRIP has started, I don’t think our demographics have changed all too much, but we’re hoping to make some progress here.” CC*

### Implementation climate: relative priority

The relative priority of TRIP amidst high existing caseloads and limited staff challenged implementation efforts. Patient navigators noted that they had large caseloads that they were required to manage. In light of these responsibilities, some viewed TRIP as “adding on” additional forms and tasks, increasing per-patient time demands, and affecting their ability to track, follow-up with, and meet the needs of all their patients.

*“I wouldn’t say it was working too well for me. We have an established program that has been around for the past 14 years. We got used to doing things a certain way… And then TRIP has changed things a little bit. It had been very difficult to adjust to it. I honestly just see it as a side task that I have to do for some patients, but I don’t see it as the standard for all of our patients… What I think is complicated [about TRIP] is adding more patients to my caseload and doing that for more and more patients. The number of patients is what’s challenging, because that’s adding on to a caseload that I already have in place...” PN*

*“I do think that it has been a lot of work for our navigators because it’s outside of their usual, standard way of doing things. And I think that TRIP, like the surveys and reconnecting with patients, at several intervals of time, has been added work for the navigators.” CC*

### Intervention characteristics: adaptability

Participants cited TRIP’s adaptability in implementing specific components as critical to successful adoption in some settings. The TRIP program allowed for flexibility within the intervention that empowered participating sites to implement TRIP components using diverse tools. For example, one clinical site already had a rigorous social risk screening and referral program in place that they used instead of using the screening and referral resource provided by TRIP. This reduction in duplicative effort facilitated the successful implementation of TRIP components at this particular institution:

*“That’s something that we talked about with the TRIP team, because the navigator in the breast clinic… is required to complete [site’s own social risk] screening for all patients. So, [it] would be just too much for her to have to complete two different social needs assessments for patients...[W]e decided to just go with [site’s social risk screen] instead of [the TRIP social risk screen].” PN*

### Intervention characteristics: complexity

Intervention-related documentation, such as tracking of TRIP patients in the shared registry, added complexity to patient navigation tasks that was viewed as a barrier to the adoption of all TRIP components. Some participants noted a misalignment between TRIP workflows versus those already established at their clinical sites. Patient navigators noted that having to complete patient data entry across multiple interfaces added additional work for patient navigators to complete which simultaneously took away from patient interaction time.

*“[TRIP is] just not going to work with us because we have to have different systems. Because our system doesn’t talk to anybody else’s so there’s always going to be duplication. That’s a real issue. I have a spreadsheet that we do for all new patients, I have a spreadsheet that I do for all of my surgery patients with my provider. And then I have a second one that my nurse practitioner put together, which is a little simpler.” PN*

*“I’ll use a scale from 1 to 100 [to rate the utility of TRIP], I guess 60%. I thought that TRIP was going to be able to support patients [with resources] and to know and learn in this process that it was much more of a documentation and communication that we were really getting.” PNS*

As noted above, the three-month social needs assessment required by TRIP in particular was perceived as counter to on-going clinical workflows and processes, and burdened patient navigators with tracking and following patients after they’ve left their care. One patient navigator working in surgery noted that they often did not work with patients at this required follow-up time point:

*“Remembering to do [the three-month follow-up social needs assessment]. [B]ecause if you’re thinking about a three-month time period… they’re pretty much gone from here by then, a lot of times. And unless somebody is seen initially and they’re doing neo-adjuvant, then I might still keep up with them during that period of time.” PN*

### Intervention characteristics: relative advantage

TRIP provided a relative advantage for many navigators in formalizing the navigation process. Participants noted that this provided legitimacy to the navigation program itself and helped guide the scope of work for navigators. In addition, the TRIP navigation protocol guidelines acted to formalize the patient navigation process. This aided patient navigators in having clear and explicit delineation of the patient navigator role within the breast cancer care clinic. Patient navigators reported having the guidelines as helpful in aiding their work.

*“The guidelines have been pretty helpful in just understanding what my role is. They outline, essentially, the minimum requirements that are expected of us as navigators in the program, and I think they’re generally just very useful.” PN*

*“[W]ithout these guidelines, especially prior to TRIP, I would have absolutely no idea what I was doing. [T]ransferring patients is definitely something that’s tricky… I guess the most difficult parts to navigate is just ensuring that if a patient is lost or getting seen elsewhere, that they are still being taken care of.” PN*

Patient navigators were also able to utilize the TRIP-provided social need screening and referral tool to find resources specific to a patient’s home location, something that they would not have been able to do from their clinic’s provided resources.

*“Sometimes if there is additional need where I feel [the TRIP-provided social needs screening and referral tool] might have more updated resources or additional things that I don’t have on our patient navigation drive. I will check it to see if there are other resources on there. There was a time where there was a patient who wanted additional resources for clothing, and I didn’t find anything that was as local as I would like it to be. I did use the [TRIP-provided] database to kind of zero in on the patient’s home address to find additional resources that were easier for her to get to, instead of having to come into the city.” PN*

### Outer setting: cosmopolitanism

The shared registry and cross-site networking allowed patient navigators to coordinate care and adopt new resources through formalizing the expansion of their networks across local clinical settings. This connection to other patient navigators was a novel aspect of the TRIP program that aided in building shared knowledge and developing a network of patient navigators that extended beyond clinical site boundaries. It was one of the mostly widely valued aspects of the TRIP intervention by patient navigators:

*“[W]ithout TRIP, I wouldn’t have been aware of other navigators at other hospitals, or had consistent contact information. [G]enerally if there was a patient who was lost to follow up, and if I had known they were going to [other clinical site], I may be able to call over to their oncology department, introduce myself and try to see if that patient was being taken care of. [T]he [shared registry] allowed me a shortcut in a way that I am able to directly reach out to somebody.” PN*

Patient navigators were able to use the shared registry system to direct message and chat with navigators at other sites, assisting with patient tracking and care coordination efforts across sites. This was perceived to improve patient care and follow-up, as well as provide navigators with an additional resource of peer colleagues to ask questions and rely upon.

*“I do think it’s helpful to communicate with other navigators at the other institutions. That’s something that we didn’t have before. I do think it’s fairly helpful when you need to send a message and transfer a patient to an outside navigator.” PN*

*“I’ve talked to other navigators when there’s been a patient who is considering care between two different institutions, or part of coordinating handoffs. I know that someone had messaged me on that platform and was just curious to know more about what navigation looked like at my institution, because they were building up navigation at their site.” PN*

*“Instead of viewing [TRIP] as a study, I do view it as more of a resource for the patients that I am meeting with, particularly those who may not be responding to phone calls or letters and seemingly have gone to different institutions." PN*

### Readiness for implementation: access to knowledge and information

Centralized structures and robust supports provided easily accessible knowledge and information for site staff as they integrated TRIP into existing workflows. The TRIP program staff assisted clinical site staff with implementation and learning each of the TRIP components, particularly while working to merge TRIP with existing workflows.

*“I think over the past year, we’ve definitely found a way to integrate it into my workflow in a way that it’s not burdensome at all.. [A]t the [start of] the study, when we were being introduced to all these different systems and expectations, [this] initially felt scary because when you’re thinking of the caseload you have now, and you’re being told there are additional expectations, it can be a little intimidating. But I would say that over the past year with the help of the TRIP team, communicating with them and giving them feedback, it’s become something that’s definitely become part of my workflow in a useful way.” PN*

Patient navigators faced a learning curve when starting TRIP activities, but this was perceived to be mitigated by TRIP administrative support and frequent follow-up and check-ins. Regular in-person site visits with patient navigators every three months, a monthly TRIP patient navigator newsletter, and weekly email check-ins were viewed as useful implementation tools by most site navigators.

*“We get regular updates. I actually got one this morning about potentially eligible patients. The newsletter is very helpful. I participate on a monthly call for the clinical folks, so the staff has been very good at keeping everyone up to date.” CC*

*“We had a lot of meetings with the TRIP team going through the guidelines. They came and presented in the breast tumor board and a lot of people were involved in that. They’re really good at communicating. They send weekly, biweekly emails, letting us know of our numbers. Just giving us any updates, we also get a newsletter. They have been very good at keeping us updated on the TRIP study numbers” PN*

## Discussion

This study assessed the implementation of the TRIP protocol originally described in Battaglia et al. 2020 [21] from the perspective of key stakeholders one year after initiation. Our analysis identified barriers and facilitators to implementing a rigorous three-part protocol and intervention for the practice of breast cancer patient navigation. The TRIP intervention was generally viewed favorably by participating sites and stakeholders. Instances where the goals and purpose of TRIP aligned with sites’ own mission and activities aided adoption of the TRIP intervention, whereas instances of misalignment hampered implementation efforts.

This rigorous 11-step protocol reflects core processes in the navigation process to empower navigators in their role addressing access and outcome disparities for breast cancer patients. The protocol utilized by TRIP to outline patient navigation activities served to formalize the patient navigation role, providing navigators with a concrete sense of their role and expectations. This structured protocol, as developed by Freund et al., 2019, helped to improve upon previously opaque expectations of the navigator role given the lack of clarity and formal definition of this position [24]. Allowing participating site staff the ability to adapt particular parts of the TRIP intervention also allowed for greater adoption and acceptance of the TRIP intervention by preventing the duplication of efforts such as using multiple tools to screen patients for unmet social needs, if those were activities already underway at a particular clinic site. The robust administrative support during and throughout the implementation process encouraged engagement with and feedback from stakeholders, leading towards the successful implementation of TRIP across sites.

A unique feature of TRIP was the creation of a city-wide navigation protocol, implemented across multiple breast cancer care institutions in the Boston area [21]. The design of the TRIP program intentionally created a shared patient registry which allowed patient navigators to communicate with peer navigators across different clinical sites, demonstrating that the TRIP program leveraged cosmopolitanism by creating structures to support cross-institutional networking. The shared registry overcame the barrier of not having interoperable electronic health record systems through establishing a communication platform that allowed for navigators to track specific patients who sought care at multiple institutions, thus aiding care coordination efforts. Patient navigators also utilized the registry to initiate contact and learn patient navigation best practices from one another, linking navigators to a professional support network that did not exist before. The registry allowed navigators to take on a “boundary spanner” role [25–28], whereby they were able to work across individual clinical site systems sites to track and aid patient care efforts in a manner that was difficult to accomplish prior to the TRIP program. Stakeholders, particularly patient navigators, noted the shared registry as one of the most impactful relative advantages offered by TRIP given the focus of prior patient navigation protocols to be specific to individual clinical sites.

Barriers were encountered given limitations of a demanding caseload and limited capacities of navigators to complete specific TRIP protocol components, such as the 3-month follow-ups with patients. Addressing these barriers requires greater institutional funding and sustainability of patient navigation efforts to bolster existing personnel resources and hire additional patient navigators, as has been demonstrated in other studies of patient navigation [29]. Given the 2016 Commission on Cancer report that mandated patient navigation programs for accreditation, many clinical sites quickly adopted patient navigation models and programs, albeit in disparate ways with wide variation in implementation and use of navigators across sites [22]. Of note, the guidelines were updated in 2020 and do not mention any requirement for a patient navigation program [30]. This change in accreditation guidelines may reduce institutional motivation to support and fund patient navigation programs through operational budgets, despite research emphasizing the benefits of patient navigation in reducing access and outcome disparities for breast cancer patients. This may serve as a broader policy impact that reduces funding and support for patient navigation programs and interventions like TRIP, in spite of the benefits of navigation as shown through research.

Our findings are limited to the perspectives of patient navigators and clinical staff at five different institutions in the Boston area and thus may not be generalizable to the practices and experiences of breast cancer patient navigation programs at healthcare institutions in different geographical areas of the United States. Notably, this study did not include patient perspectives regarding the impact of TRIP-enhanced patient navigation on breast cancer patients which is an important area of future exploration. Our data collection period was affected by a research hiatus during the initial months of the COVID-19 pandemic. Given that we were focusing on barriers and facilitators of the initial implementation of TRIP, we excluded references to changes at clinical sites processes and roles due to the ongoing pandemic. The hiatus in recruitment and data collection may have resulted in some recall bias of initial implementation efforts given the extended time period between initial implementation and the interview period.

## Conclusions

Our study describes the facilitators and barriers of implementing a standardized patient navigation protocol across multiple healthcare institutions and builds upon prior research calling for greater clarity in describing the patient navigator role [15, 31]. Implementation efforts were facilitated when both broad goals and more narrow workflows fit with or provided value to individual site programs. Understanding barriers and facilitators such as the ones identified here can aid other sites that seek to implement evidence-based navigation protocols. These findings can support other health systems interested in role addressing access and outcome disparities for breast cancer patients through patient navigation.

## Data Availability

The datasets used and/or analyzed during the current study are available from the corresponding author on reasonable request.

## Abbreviations

CFIR: Consolidated Framework for Implementation Research
CC: Clinical champion
PN: Patient navigator
PNS: Patient navigator supervisor
TRIP: Translating Research Into Practice
